# Supplementary material for: PRISMA and BioID disclose a motifs-based interactome of the intrinsically disordered transcription factor C/EBPα
Source: iScience. 2021 Jun 4;24(6):102686. doi: 10.1016/j.isci.2021.102686 (PMC8220391; doi:10.1016/j.isci.2021.102686)
Supplement: Document S1. Figures S1–S5 and Tables S1 and S2 [file mmc1.pdf]

iScience, Volume *24*

Supplemental information

PRISMA and BioID disclose a motifs-based interactome of the intrinsically disordered transcription factor C/EBPα

Evelyn Ramberger, Valeria Sapozhnikova, Elisabeth Kowenz-Leutz, Karin Zimmermann, Nathalie Nicot, Petr V. Nazarov, Daniel Perez-Hernandez, Ulf Reimer, Philipp Mertins, Gunnar Dittmar, and Achim Leutz

Position (Human) CEBPA P42	Position (rat) CEBPA P42	PTM Type	PTM included PRISMA screen	Known PTM/Reference (Human)	Known PTM/Reference (Rat)	known PTM features	BioID detected	phenotype
R12	R12	methylation	me2sym, me2asym				me, me2	
S21	S21	phos	phos	PMID:20101026,	PMID:26515730, 16807249	phos	phos	disrupts granulocytic differentiation
R35	R35	methylation	me2sym, me2asym	PMID:31015230		me		increased cyclin D1 expression and proliferation
K86	K86	acetylation methylation	ac,me2					
K90	K90	acetylation methylation	ac,me2					
K92	K92	acetylation methylation	ac,me2					
R142	R140	methylation	me2sym, me2asym				me	
R149	R147	methylation	me2sym, me2asym					
R156	R154	methylation	me2sym, me2asym	PMID:31015230		me, me2	me	increased cyclin D1 expression and proliferation
K161	K159	methylation acetylation ubiquitination sumoylation	ac, me2	PMID:19608861 PMID:21890473	https://doi.org/10.18452/17281 PMID:22902405 PMID:23639777	me ac ub sumo		interaction with HDAC3
R165	R163	citrullination methylation	citr, me2sym, me2asym		https://doi.org/10.18452/17281	citr		
K171	K169	acetylation methyalation	ac, me2					
S190	S193	phos	phos	PMID:27619993, 2615376, 15107404		phos		growth inhibition
T226	T222	phos	phos	PMID:16600022	PMID:26153766 , 27619993 , 16600022	phos		regulation of lipogenic and gluconeogenic gene expression
T230	T226	phos	phos	PMID:16600022	PMID:16600022	phos		regulation of lipogenic and gluconeogenic gene expression
S234	S230	phos	phos	PMID:20101026	PMID:17290224, 10567568	phos		
K254	K250	acetylation methylation	ac,me2					
R264	R260	methylation	me2sym, m2asym					
S266	G262	phos	phos	PMID:25159151		phos		
K352	K352	acetylation methylation	ac,me2					

Table S1: CEBPA PTMs included in PRISMA, related to Figure 1

#	sequence	unmodified sequence	region	AA start	AA end	modification	number interactors
1	MESADFYEAEPRPPM	MESADFYEAEPRPPM	CR2	1	15		284
2	M-Nterm.ac-ESADFYEAEPRPPM	MESADFYEAEPRPPM	CR2	1	15	('1', 'N-term ac')	265
3	MESADFYEAEPR-me2_sym-PPM	MESADFYEAEPRPPM	CR2	1	15	('12', 'me2_sym')	231
4	MESADFYEAEPR-me2_asym-PPM	MESADFYEAEPRPPM	CR2	1	15	('12', 'me2_asym')	223
5	EAEPRPPMSSHLQSP	EAEPRPPMSSHLQSP		8	22		1
6	EAEPR-me2_sym-PPMSSHLQSP	EAEPRPPMSSHLQSP		8	22	('12', 'me2_sym')	2
7	EAEPRPPMSSHLQS-phos-P	EAEPRPPMSSHLQSP		8	22	('21', 'phos')	6
8	EAEPR-me2_asym-PPMSSHLQSP	EAEPRPPMSSHLQSP		8	22	('12', 'me2_asym')	14
9	MSSHLQSPPHAPSSA	MSSHLQSPPHAPSSA		15	29		0
10	MSSHLQS-phos-PPHAPSSA	MSSHLQSPPHAPSSA		15	29	('21', 'phos')	0
11	PPHAPSSAAFGFPRG	PPHAPSSAAFGFPRG		22	36		0
12	PPHAPSSAAFGFPR-me2_sym-G	PPHAPSSAAFGFPRG		22	36	('35', 'me2_sym')	1
13	PPHAPSSAAFGFPR-me2_asym-G	PPHAPSSAAFGFPRG		22	36	('35', 'me2_asym')	0
14	AAFGFPRGAGPAQPP	AAFGFPRGAGPAQPP		29	43		14
15	AAFGFPR-me2_sym-GAGPAQPP	AAFGFPRGAGPAQPP		29	43	('35', 'me2_sym')	17
16	AAFGFPR-me2_asym-GAGPAQPP	AAFGFPRGAGPAQPP		29	43	('35', 'me2_asym')	15
17	GAGPAQPPAPPAAPE	GAGPAQPPAPPAAPE		36	50		1
18	PAPPAAPEPLGGICE	PAPPAAPEPLGGICE	CR3	43	57		1
19	EPLGGICEHETSIDI	EPLGGICEHETSIDI	CR3	50	64		48
20	EHETSIDISAYIDPA	EHETSIDISAYIDPA	CR3	57	71		106
21	ISAYIDPAAFNDEFL	ISAYIDPAAFNDEFL	CR3	64	78		296
22	AAFNDEFLADLFQHS	AAFNDEFLADLFQHS	CR4	71	85		301
23	LADLFQHSRQQEKAK	LADLFQHSRQQEKAK		78	92		0
24	LADLFQHSRQQEK-ac-AK	LADLFQHSRQQEKAK		78	92	('90', 'ac')	8
25	LADLFQHSR-me2_asym-QQEKAK	LADLFQHSRQQEKAK		78	92	('86', 'me2_asym')	0
26	LADLFQHSR-me2_sym-QQEKAK	LADLFQHSRQQEKAK		78	92	('86', 'me2_sym')	1
27	LADLFQHSRQQEK-me2-AK	LADLFQHSRQQEKAK		78	92	('90', 'me2')	0
28	SRQQEKAKAAVGPTG	SRQQEKAKAAVGPTG		85	99		0
29	SRQQEKAK-me2-AAVGPTG	SRQQEKAKAAVGPTG		85	99	('92', 'me2')	0
30	SR-me2_asym-QQEKAKAAVGPTG	SRQQEKAKAAVGPTG		85	99	('86', 'me2_asym')	0
31	SR-me2_sym-QQEKAKAAVGPTG	SRQQEKAKAAVGPTG		85	99	('86', 'me2_sym')	0
32	SRQQEK-ac-AKAAVGPTG	SRQQEKAKAAVGPTG		85	99	('90', 'ac')	0
33	SRQQEKAK-ac-AAVGPTG	SRQQEKAKAAVGPTG		85	99	('92', 'ac')	0
34	SRQQEK-me2-AKAAVGPTG	SRQQEKAKAAVGPTG		85	99	('90', 'me2')	0
35	K-ac-AAVGPTGGGGGGDF	KAAVGPTGGGGGGDF		92	106	('92', 'ac')	11
36	GGGGGGDFDYPGAPA	GGGGGGDFDYPGAPA	CR5	99	113		33
37	FDYPGAPAGPGGAVM	FDYPGAPAGPGGAVM		106	120		7
38	AGPGGAVMPGGAHGP	AGPGGAVMPGGAHGP		113	127		5
39	MPGGAHGPPPGYGCA	MPGGAHGPPPGYGCA		120	134		14
40	M-Nterm.ac-PGGAHGPPPGYGCA	MPGGAHGPPPGYGCA		120	134	('120', 'N-term ac')	8
41	PPPGYGCAAAGYLDG	PPPGYGCAAAGYLDG	CR1L	127	141		16
42	AAAGYLDGRLEPLYE	AAAGYLDGRLEPLYE	CR1L	134	148		172
43	AAAGYLDGR-me2_asym-LEPLYE	AAAGYLDGRLEPLYE	CR1L	134	148	('142', 'me2_asym')	205
44	AAAGYLDGR-me2_sym-LEPLYE	AAAGYLDGRLEPLYE	CR1L	134	148	('142', 'me2_sym')	271
45	GRLEPLYERVGAPAL	GRLEPLYERVGAPAL	CR1L	141	155		6
46	GR-me2_asym-LEPLYERVGAPAL	GRLEPLYERVGAPAL	CR1L	141	155	('142', 'me2_asym')	9
47	GR-me2_sym-LEPLYERVGAPAL	GRLEPLYERVGAPAL	CR1L	141	155	('142', 'me2_sym')	2
48	GRLEPLYER-me2_asym-VGAPAL	GRLEPLYERVGAPAL	CR1L	141	155	('149', 'me2_asym')	5
49	GRLEPLYER-me2_sym-VGAPAL	GRLEPLYERVGAPAL	CR1L	141	155	('149', 'me2_sym')	1
50	ERVGAPALRPLVIKQ	ERVGAPALRPLVIKQ		148	162		0
51	ER-me2_asym-VGAPALRPLVIKQ	ERVGAPALRPLVIKQ		148	162	('149', 'me2_asym')	1
52	ERVGAPALR-me2_sym-PLVIKQ	ERVGAPALRPLVIKQ		148	162	('156', 'me2_sym')	3
53	ERVGAPALR-me2_asym-PLVIKQ	ERVGAPALRPLVIKQ		148	162	('156', 'me2_asym')	1
54	ERVGAPALRPLVIK-me2-Q	ERVGAPALRPLVIKQ		148	162	('161', 'me2')	0
55	ERVGAPALRPLVIK-ac-Q	ERVGAPALRPLVIKQ		148	162	('161', 'ac')	1
56	LRPLVIKQEPREEDE	LRPLVIKQEPREEDE	CR6	155	169		8
57	LR-me2_sym-PLVIKQEPREEDE	LRPLVIKQEPREEDE	CR6	155	169	('156', 'me2_sym')	31
58	LR-me2_asym-PLVIKQEPREEDE	LRPLVIKQEPREEDE	CR6	155	169	('156', 'me2_asym')	19
59	LRPLVIKQEPR-me2_sym-EEDE	LRPLVIKQEPREEDE	CR6	155	169	('164', 'me2_sym')	26
60	LRPLVIK-me2-QEPREEDE	LRPLVIKQEPREEDE	CR6	155	169	('161', 'me2')	16

61	LRPLVIK-ac-QEPREEDE	LRPL**VIKQEPREEDE**E	**CR6**	155	169	('161', 'ac')	47
62	LRPLVIKQEPR-me2_asym-EEDE	LRPL**VIKQEPREEDE**E	**CR6**	155	169	('164', 'me2_asym')	12
63	LRPLVIKQEPR-citr-EEDE	LRPL**VIKQEPREEDE**E	**CR6**	155	169	('164', 'citr')	53
64	QEPREEDEAK-me2-QLALA	**QEPREED**EAKQLALA		162	176	('171', 'me2')	26
65	QEPREEDEAK-ac-QLALA	**QEPREED**EAKQLALA		162	176	('171', 'ac')	41
66	QEPR-me2_asym-EEDEAKQLALA	**QEPREED**EAKQLALA		162	176	('164', 'me2_asym')	6
67	QEPR-citr-EEDEAKQLALA	**QEPREED**EAKQLALA		162	176	('164', 'citr')	26
68	EAKQLALAGLFPYQP	EAKQLALAGLFPYQP		169	183		2
69	EAK-ac-QLALAGLFPYQP	EAKQLALAGLFPYQP		169	183	('171', 'ac')	8
70	EAK-me2-QLALAGLFPYQP	EAKQLALAGLFPYQP		169	183	('171', 'me2')	1
71	AGLFPYQPPPPPPPS	AGLFPYQPPPPPPPS		176	190		37
72	PPPPPPPSHPHPHPP	PPPPPPPSHPHPHPP		183	197		7
73	PPPPPPPS-phos-HPHPHPP	PPPPPPPSHPHPHPP		183	197	('190', 'phos')	1
74	SHPHPHPPPAHLAAP	SHPHPHPPPAHLAAP		190	204		0
75	S-phos-HPHPHPPPAHLAAP	SHPHPHPPPAHLAAP		190	204	('190', 'phos')	0
76	PPAHLAAPHLQFQIA	PPAHLAA**PHLQFQIA**	**CR7**	197	211		2
77	PHLQFQIAHCGQTTM	**PHLQFQIAHCGQTTM**	**CR7**	204	218		0
78	AHCGQTTMHLQPGHP	**AHCGQTTMHLQPGHP**	**CR7**	211	225		0
79	MHLQPGHPTPPPTPV	**MHLQPGHPTPPPTPV**	**CR7**	218	232		1
80	MHLQPGHPT-phos-PPPTPV	**MHLQPGHPTPPPTPV**	**CR7**	218	232	('226', 'phos')	2
81	MHLQPGHPTPPPT-phos-PV	**MHLQPGHPTPPPTPV**	**CR7**	218	232	('230', 'phos')	4
82	PTPPPTPVPSPHPAP	**PTPPPTPVP**SPHPAP	**CR7**	225	239		2
83	PT-phos-PPPTPVPSPHPAP	**PTPPPTPVP**SPHPAP	**CR7**	225	239	('226', 'phos')	0
84	PTPPPT-phos-PVPSPHPAP	**PTPPPTPVP**SPHPAP	**CR7**	225	239	('230', 'phos')	1
85	PTPPPTPVPS-phos-PHPAP	**PTPPPTPVP**SPHPAP	**CR7**	225	239	('234', 'phos')	0
86	VPSPHPAPALGAAGL	VPSPHPAPALGAAGL		232	246		1
87	VPS-phos-PHPAPALGAAGL	VPSPHPAPALGAAGL		232	246	('234', 'phos')	0
88	PALGAAGLPGPGSAL	PALGAAGLPGPGSAL		239	253		1
89	LPGPGSALKGLGAAH	LPGPGSALKGLGAAH		246	260		0
90	LPGPGSALK-ac-GLGAAH	LPGPGSALKGLGAAH		246	260	('254', 'ac')	0
91	LPGPGSALK-me2-GLGAAH	LPGPGSALKGLGAAH		246	260	('254', 'me2')	0
92	LKGLGAAHPDLRASG	LKGLGAAHPDLRASG		253	267		0
93	LK-me2-GLGAAHPDLRASG	LKGLGAAHPDLRASG		253	267	('254', 'me2')	0
94	LK-ac-GLGAAHPDLRASG	LKGLGAAHPDLRASG		253	267	('254', 'ac')	0
95	LKGLGAAHPDLRAS-phos-G	LKGLGAAHPDLRASG		253	267	('266', 'phos')	0
96	LKGLGAAHPDLR-me2_sym-ASG	LKGLGAAHPDLRASG		253	267	('264', 'me2_sym')	5
97	LKGLGAAHPDLR-me2_asym-ASG	LKGLGAAHPDLRASG		253	267	('264', 'me2_asym')	0
98	HPDLRASGGSGAGKA	HPDLRASGGSGAGKA		260	274		0
99	HPDLR-me2_asym-ASGGSGAGKA	HPDLRASGGSGAGKA		260	274	('264', 'me2_asym')	0
100	HPDLRAS-phos-GGSGAGKA	HPDLRASGGSGAGKA		260	274	('266', 'phos')	0
101	HPDLR-me2_sym-ASGGSGAGKA	HPDLRASGGSGAGKA		260	274	('264', 'me2_sym')	0
102	GGSGAGKAKKSVDKN	GGSGA**GKAKKSVDKN**	**BZIP**	267	281		0
103	AKKSVDKNSNEYRVR	**AKKSVDKNSNEYRVR**	**BZIP**	274	288		0
104	NSNEYRVRRERNNIA	**NSNEYRVRRERNNIA**	**BZIP**	281	295		5
105	RRERNNIAVRKSRDK	**RRERNNIAVRKSRDK**	**BZIP**	288	302		126
106	AVRKSRDKAKQRNVE	**AVRKSRDKAKQRNVE**	**BZIP**	295	309		243
107	KAKQRNVETQQKVLE	**KAKQRNVETQQKVLE**	**BZIP**	302	316		1
108	ETQQKVLELTSDNDR	**ETQQKVLELTSDNDR**	**BZIP**	309	323		2
109	ELTSDNDRLRKRVEQ	**ELTSDNDRLRKRVEQ**	**BZIP**	316	330		1
110	RLRKRVEQLSRELDT	**RLRKRVEQLSRELDT**	**BZIP**	323	337		1
111	QLSRELDTLRGIFRQ	**QLSRELDTLRGIFRQ**	**BZIP**	330	344		1
112	TLRGIFRQLPESSLV	**TLRGIFRQLPE**SSLV	**BZIP**	337	351		1
113	QLPESSLVKAMGNCA	**QLPE**SSLVKAMGNCA		344	358		0
114	QLPESSLVK-me2-AMGNCA	**QLPE**SSLVKAMGNCA		344	358	('352', 'me2')	0

Table S2: CEBPA peptides screened for interactions with PRISMA, related to Figure 1 and STAR methods

List of peptides included in the PRISMA screen. Membership of peptide sequence to CEBPA conserved region (CR) is indicated and CR sequences (Leutz et al., 2011) are highlighted in color. BZIP = basic leucine zipper

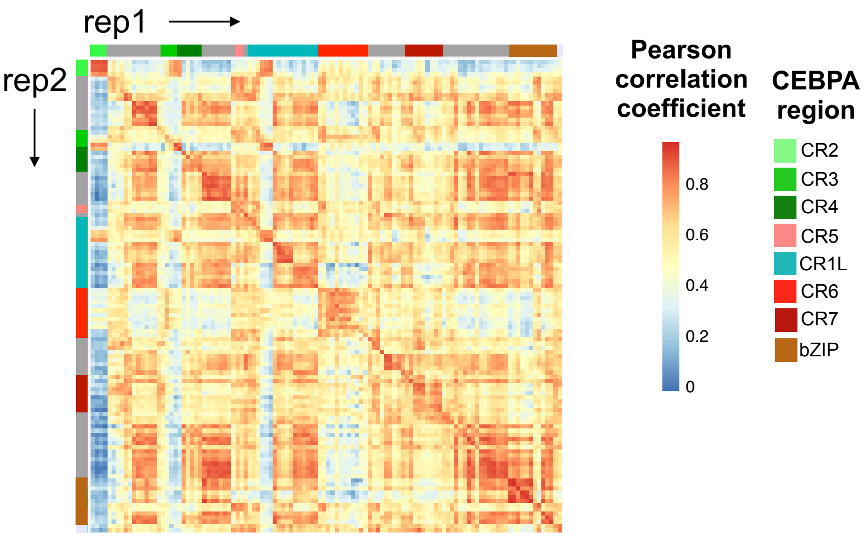

Figure S1: Pearson correlation matrix of PRISMA replicates, Related to Figure 1

Pearson correlation of log2 LFQ values in each PRISMA spot (114 spots). Replicate 1 is plotted against replicate 2. Annotation bars indicates conserved regions of CEBPA.

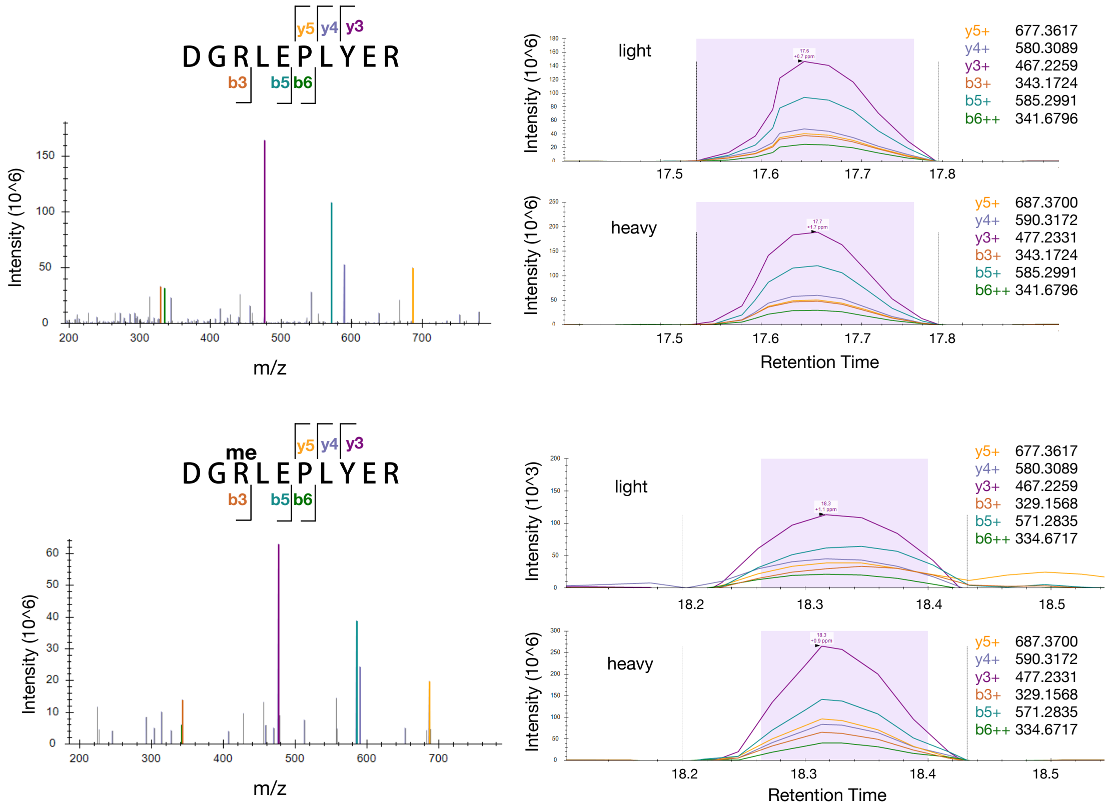

Figure S2: CEBPA is methylated at R142, Related to Figure 1 and 3

Parallel reaction monitoring (PRM) of unmodified (top) and methylated (bottom) peptides spanning R142. A heavy peptide standard isotopically labeled at the C-terminal arginine was used to confirm the identity of the peptide. Left: MS2 spectrum of heavy peptide standard; right: elution profile of MS2 fragments of light and heavy peptides. PRM data was analyzed with Skyline

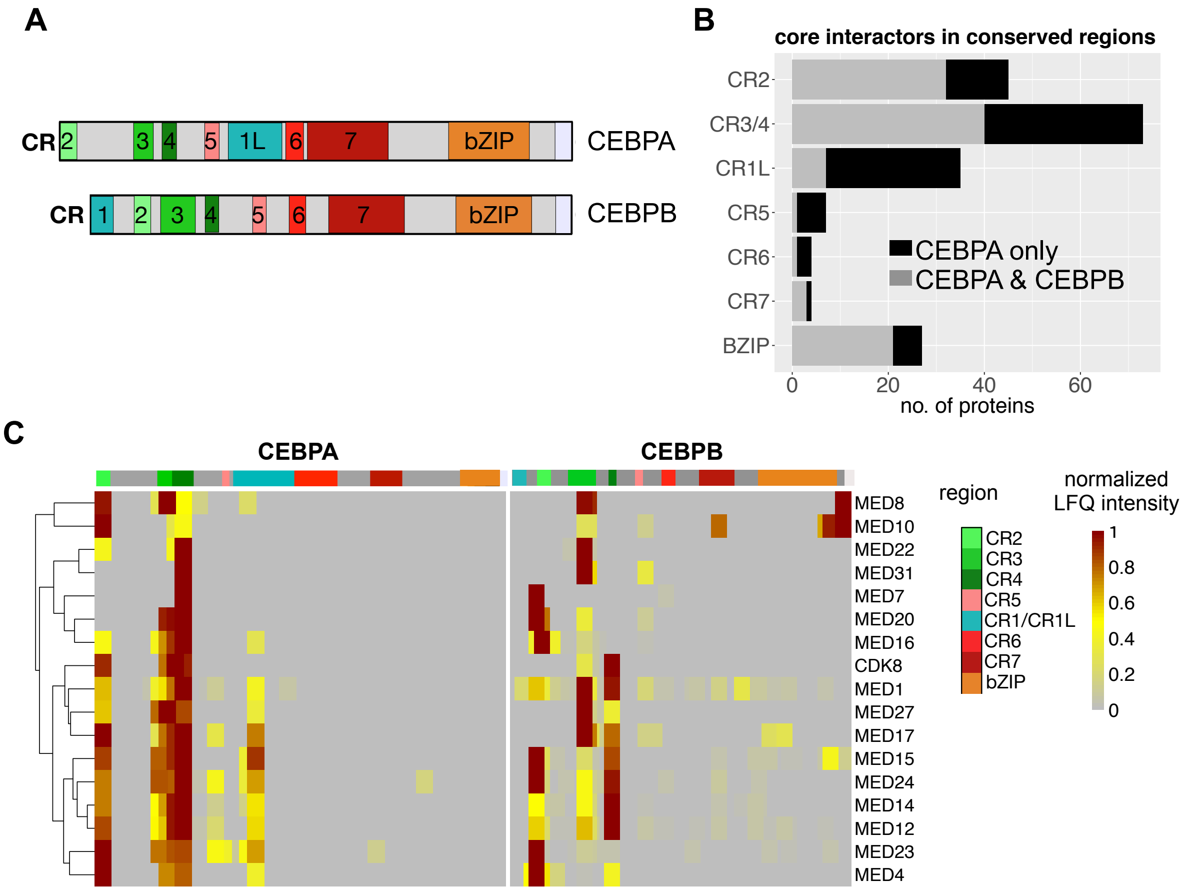

Figure S3: CEBPA and CEBPB share interactors in homologous regions, Related to Figure 2
A: Shared homology of conserved regions in CEBPA and CEBPB. B: Number of high-confidence CEBPA interactors per conserved region in CEBPA as detected by PRISMA and BioID (black bars). Grey bars represent interactors that were also identified as interactors in homologues regions in CEBPB (Dittmar et al., 2019). C: Extracted PRISMA binding profiles of Mediator complex subunits binding to CEBPA and CEBPB (right). Annotation bar on top indicates conserved regions

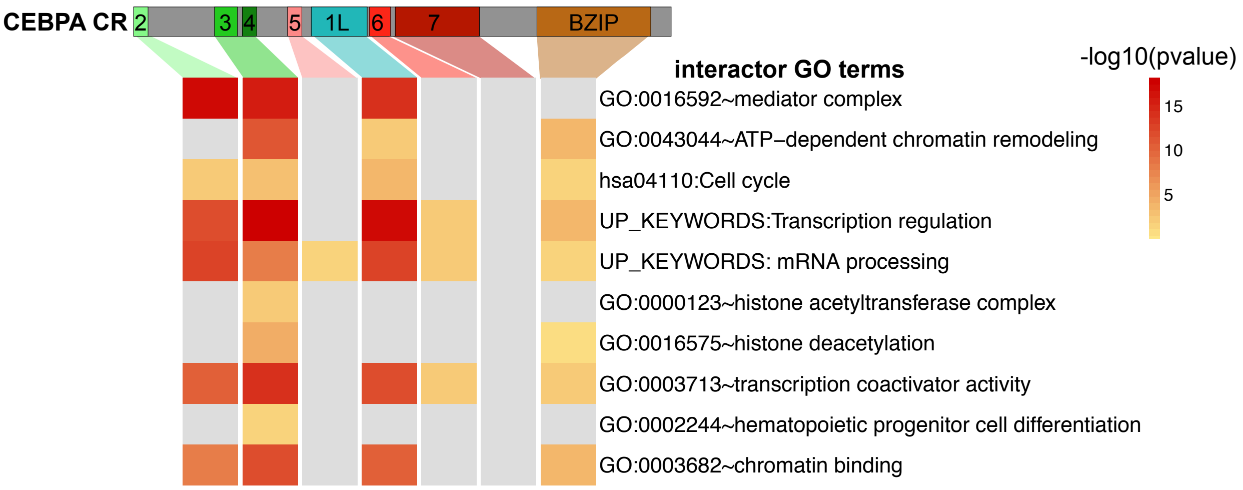

Figure S4: GO term enrichment of mapped CEBPA interactors, Related to Figure 2
PRISMA CEBPA interactors confirmed by BioID were subjected to GO term and domain analysis using the DAVID tool. Informative significant GO terms (p value < 0.05) are displayed. Grey indicates no significant enrichment. No GO terms were significantly enriched in CR7 binders. A complete list of all significant GO terms and domains is available in Table S5.

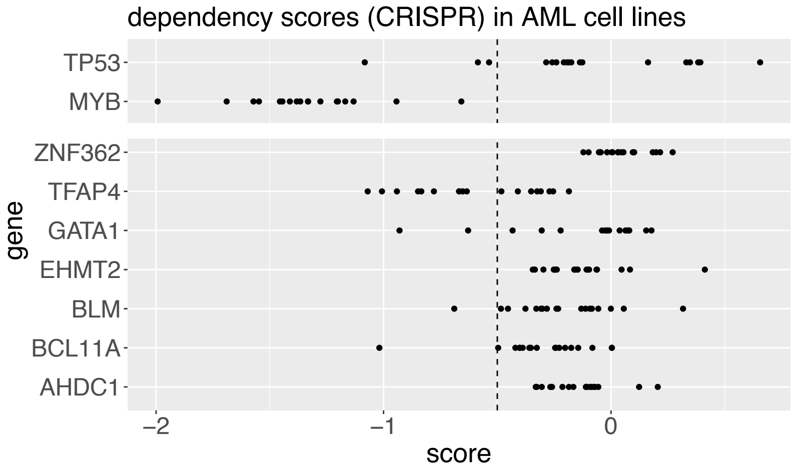

Figure S5: CEBPA P30 specific interactors may represent therapeutic targets in AML, related to Figure 4
Dependency scores from CRISPR knockout screens in AML cell lines extracted from the DepMap portal. Scores of P30 specific interactors are displayed. Known tumor-suppressor P53 and oncogene MYB are plotted on top as a reference.
